# Long-axial field-of-view PET/CT: perspectives and review of a revolutionary development in nuclear medicine based on clinical experience in over 7000 patients

**DOI:** 10.1186/s40644-023-00540-3

**Published:** 2023-03-18

**Authors:** Ian Alberts, Hasan Sari, Clemens Mingels, Ali Afshar-Oromieh, Thomas Pyka, Kuangyu Shi, Axel Rominger

**Affiliations:** 1 Department of Nuclear Medicine, Inselspital, Bern University Hospital, University of Bern, Freiburgstr. 18, 3010 Bern, Switzerland; 2 Advanced Clinical Imaging Technology, Siemens Healthcare AG, Lausanne, Switzerland

**Keywords:** Total-body, Long axial field of view (LAFOV) PET, Whole-body, PET/CT, Positron emission tomography, Digital PET

## Abstract

Recently introduced long-axial field-of-view (LAFOV) PET/CT systems represent one of the most significant advancements in nuclear medicine since the advent of multi-modality PET/CT imaging. The higher sensitivity exhibited by such systems allow for reductions in applied activity and short duration scans. However, we consider this to be just one small part of the story: Instead, the ability to image the body in its entirety in a single FOV affords insights which standard FOV systems cannot provide. For example, we now have the ability to capture a wider dynamic range of a tracer by imaging it over multiple half-lives without detrimental image noise, to leverage lower radiopharmaceutical doses by using dual-tracer techniques and with improved quantification. The potential for quantitative dynamic whole-body imaging using abbreviated protocols potentially makes these techniques viable for routine clinical use, transforming PET-reporting from a subjective analysis of semi-quantitative maps of radiopharmaceutical uptake at a single time-point to an accurate and quantitative, non-invasive tool to determine human function and physiology and to explore organ interactions and to perform whole-body systems analysis. This article will share the insights obtained from 2 years’ of clinical operation of the first Biograph Vision Quadra (Siemens Healthineers) LAFOV system. It will also survey the current state-of-the-art in PET technology. Several technologies are poised to furnish systems with even greater sensitivity and resolution than current systems, potentially with orders of magnitude higher sensitivity. Current barriers which remain to be surmounted, such as data pipelines, patient throughput and the hindrances to implementing kinetic analysis for routine patient care will also be discussed.

## Introduction

The use of positron emitting radiopharmaceuticals for the imaging of human function dates back to pioneering work in the 1950’s [1, 2] and has, over the course of this long history, undergone a number of revolutionary developments. Examples include the tremendously successful introduction of 2-[^18^F]FDG in 1976 [3, 4], the first single ring PET scanner [5], the first positron emission tomograph in 1975 [6] and the development of three-dimensional PET systems [5]. Although the terms “game-changing” or “paradigm shift” have been overused to the point of cliché, nuclear medicine has genuinely experienced several disruptive changes that have fundamentally changed the field in recent decades. One such profound change was the successful introduction of multimodal PET/CT imaging at the turn of the twenty-first century [7]. This catalysed the field. Presently, PET/CT imaging occupies a central role in the staging and management of a number of cancers [8] as well an increasing role in numerous non-oncological indications. The challenges and complexities of delivering hybrid functional and anatomical imaging has had profound implications for the training and organisation of our field [9–14].

The detectors used in PET/CT systems have long been comprised of scintillation crystals coupled with conventional photomultiplier tubes (PMT). Replacement of this analogue technology with solid state detectors based on silicon photomultipliers (SiPM) was an important recent development in PET imaging; these systems offer a number of technical advantages which overcome the limitations of previous analogue systems [15]. In particular, the greatly improved time-of-flight (TOF) resolution enables noise reduction and TOF effective sensitivity gains, resulting in improved clinical performance [16–21], including better image quality and lesion detection [15, 20, 22, 23]. The first positron imaging systems comprised of a single ring of detectors. Cognisant of the fact that scanner sensitivity and image noise are important limiting factors in PET imaging, scanners have increasingly been designed with longer axial field-of-views (aFOV), such as the Biograph mCT (Siemens Healthineers, aFOV of 22 cm), Biograph Vision 600 (Siemens Healthineers, aFOV of 26.3 cm) [15] and the recently described GE Discovery MI PET/CT system (General Electric, aFOV of 30 cm) [24]. The combination of solid state detectors with increased aFOV coverage represents an important improvement upon the previous generation of scanners and demonstrates an increased photon sensitivity and peak noise equivalent count rate (NECR) [25]. However, such systems are still limited by the restriction of their FOV to one bed position, requiring continuous bed motion (CBM) for full-body coverage. A substantial leap forward was made with the realisation of extended FOV PET systems, such as the PennPET Explorer (up to 140 cm aFOV) and the uExplorer (United Imaging, 194 cm aFOV) as well as the Biograph Vision Quadra as described earlier [26–28]. The design of these systems is based on pioneering work which surmounted a number of technical challenges [25, 27, 29]. Rather than representing an incremental improvement, these systems can be understood to represent a new, truly revolutionary design. For the first time, it is now possible to image the head and entire trunk simultaneously in adult human subjects, furnishing new and unique insights into human function and physiology. It is our position that this represents an entirely new way of performing PET and promises to be as revolutionary a change to the field as the introduction of hybrid imaging.

## Challenges and opportunities

Our group received and operated the world’s first clinical Biograph Vision Quadra (Siemens Healthineers) system at the Department for Nuclear Medicine, Inselspital in Bern, Switzerland in October 2020 (https://www.swissinfo.ch/eng/world-s-fastest-full-body-scanner-turned-on-in-bern/46184876). Building upon the pioneering PennPET and uExplorer, the Biograph Vision Quadra (106 cm aFOV) occupies a middle-ground between standard FOV and total-body systems, capable of imaging the head to the mid-thighs for adult subjects in a single bed-position [30–32] and where sensitivity does not substantially improve beyond 100 cm, proving an economical use of space and detector material [25, 27, 29]. The footprint of this particular system also has the advantage that it can be installed into an examination room with the same dimensions as for a standard axial FOV (SAFOV) system, meaning that no complex or expensive infrastructure adjustments were necessary. A number of terms such as whole-body, total-body, extended and ultra-extended FOV have been used to describe these systems, which for simplicity we henceforth refer to as long-axial FOV (LAFOV) systems in contradistinction to SAFOV systems, which we take to mean systems which can image only a part of the body in a single bed position.

The challenges of assembling a multinational team of scientists and engineers to install this complex system during the height of the COVID-19 pandemic notwithstanding, the scanner went live in October 2020; some 7000 patients have now been scanned at our centre with this system. The aim of this article is to share our experiences operating the first Biograph Vision Quadra system and the insights we have gained.

### Operational considerations and patient throughput in LAFOV scanning

With a steadily increasing number of clinical indications for PET/CT imaging, demand for PET services are increasing, adding to pressure on waiting lists [33]. There is therefore a clear rationale for increasing patient throughput in many PET/CT centres, and the ability to scan more patients with less activity is an attractive prospect. A standard acquisition at 2 min per bed position can take between 15 and 20 min on a SAFOV system depending on body length, although it is well worth noting that this is already very fast compared to other imaging modalities, such as MRI, and is substantially faster than the acquisition times used on first generation PET/CT systems. Nevertheless the time taken per scan does potentially represent a bottle-neck in the number of scans which can be performed. With a LAFOV system, a comparable acquisition can be obtained in just 2 min when using standard activities, with the possibility for maintaining adequate image quality in as short as 30s [31]. Theoretically, allowing an additional 8 min for getting the patient on and off the examination table, 60 patients could be imaged on a single LAFOV scanner in the course of a 10 h working day, although additional acquisitions such as contrast enhanced CT could take additional time. Given that the optimal number of uptake rooms is the ratio of uptake time to scan time [34], theoretically only six uptake rooms or hot waiting areas would be needed, ideally with additional changing rooms to optimise patient flow. However, this would be a significant increase in patient throughput for most centres, and many more personnel would be needed to realise this demanding timetable. Beyond operational consideration, there are also compelling arguments for shorter scan durations [35] such as reduction in motion artefact and patient comfort, although the impact of this on image quality is yet to be assessed. The premise of short duration protocols is also the use of standard activities; instead, the higher sensitivity can also be exploited to reduce dose, as will be discussed later in this article.

At our centre, we have opted to balance quality and speed by providing patients with 6 min total scan time, which is both shorter than a standard acquisition on a SAFOV system and with substantially superior count rates [31]. For paediatric patients, we routinely inject a lower activity of 0.5-1.0 MBq/kg (substantially lower than EANM/SNMMI guidelines [36]). This is combined with a 10 min acquisition, already shorter than an equivalent SAFOV scan in many cases and resulting in a “win-win” scenario when imaging children. At other centres, ultra-low activity and short scans which obviate the need for sedation or anaesthesia have also been reported, improving patient flow and patient safety [37].

### Improving the view: whole-body imaging

**Fig. 1:**
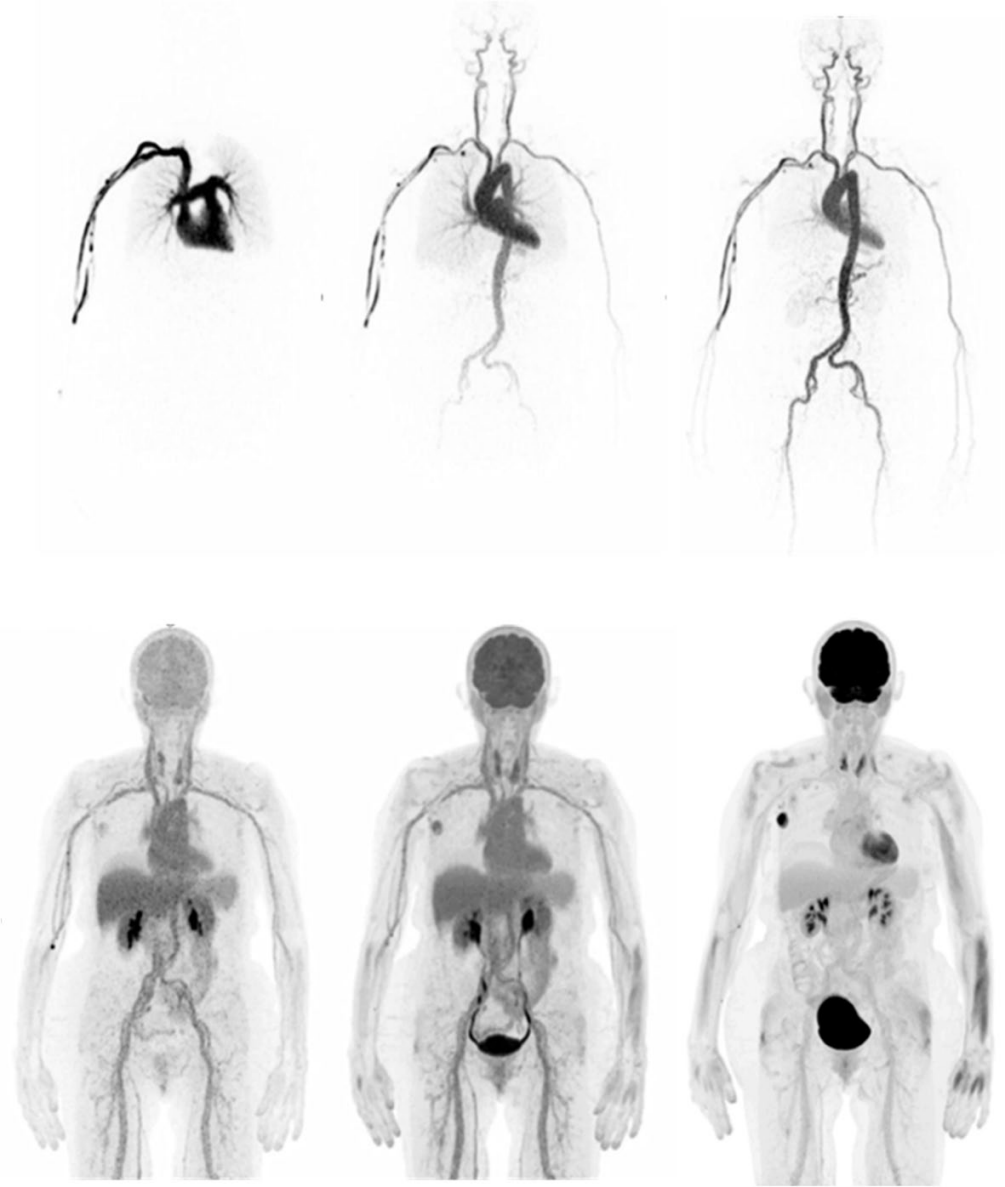
Selected time frames for a patient examined at the University Hospital, Bern with breast cancer (top row between 0-60s p.i.; bottom row circa 5 min, 30 min and final static scan at 55–65 min) demonstrate the excellent temporal and spatial resolution offered by LAFOV systems. The radiopharmaceutical (2-[^18^F]FDG) can be traced through the pulmonary vasculature, arteries and venous system with small to medium sized vessels visible

In common with most centres, our SAFOV imaging protocol captures the skull base to the mid-thigh, where restriction of the examination volume allows the scan to be completed faster. One advantage of the uExplorer is the ability to capture the entirety of the human organism from head to toe; other LAFOV systems can capture the head and torso in a single FOV. This ability to image the entire torso represents a significant advantage, for example when exploring whole-body organ interactions [38] or to enable whole-body, multi-organ kinetic imaging [39] which potentially reveals insights which previous generation SAFOV could not provide. Figure 1 shows an example of a patient acquired using a dynamic imaging protocol with our LAFOV system and demonstrates the high-temporal resolution and low-noise whole-body dynamic data which can be achieved using LAFOV systems.

Although some high volume centres may be able to make full use of a LAFOV scanner’s additional capacity and while there are a number of compelling arguments for activity reduction, it is our view that this is only one small part of the LAFOV story. Indeed, although reducing the applied activity by 50% is laudable [40], high-sensitivity digital PET/CT systems were already able to provide similar reductions [18, 20, 21, 23]. Moreover, we find that this focus on activity and time does not fully leverage the benefits that high-quality and total-body data might deliver. Fundamentally, a lower-activity scan presented in the same static standardised uptake value (SUV) map with marginally lower image noise, while an improvement, does not necessarily represent a quantum leap into previously unknown technology or afford any new insight when compared to imaging with a SAFOV system using SiPM [41]. Instead, high-count statistic total body imaging affords the ability to probe whole-body pharmacokinetic data [39, 42–44] and organ-organ interactions [38] in a way that the physics of a SAFOV system could not allow [45, 46]. In our view, it is these previously hidden insights that are the most exciting and promising aspect of LAFOV systems, and one which has the potential to revolutionise the way in which PET/CT imaging is performed. In this article, we will share some of our insights and experiences in using this new technology and explore some novel directions for research which might be performed with this scanner, as well as some of the challenges we have encountered.

### Managing LAFOV data

The substantially higher count densities result in increased data volumes, which can be as high as 4-5GB for a standard SAFOV scan and as high as 40-50 GB for a LAFOV scan when the PET list-mode raw data is stored and depending on activity, scan length and additional PET data such as deep inspiratory or triggered acquisitions. One hour long dynamic acquisitions are larger yet, and can consume between 500GB and a terabyte of storage. For day-to-day clinical purposes, these data sizes have no impact on the archiving and viewing of clinical images if only secondary static reconstructions are stored in clinical archiving systems (PACS). However, it is often the case that when performing research, storage and subsequent retrieval of the full raw data set is needed for research specific reconstructions or analysis of the raw data. Since LAFOV systems lend themselves well to whole-body dynamic imaging, the problem of storage of raw data-sets is likely to be encountered by many users of this system. By way of example, in our first two years of operation, storage of the list-mode for our single LAFOV PET alone consumed almost 4.5 petabytes of data-storage; by comparison the ATLAS project at CERN, one of the most complex and data intensive experiments yet devised by mankind requires 10 petabytes of data storage per annum and is supported by a team of 5700 scientists, engineers and administrators with access to one of the world’s most advanced IT infrastructures (https://atlas.cern/Resources/Fact-sheets). Storage of such data volumes is a fundamental change by many orders of magnitude compared to previous generation systems, e.g. the storage of raw data for our two predecessor analogue Siemens Biograph mCT systems over several years of clinical operation generated approximately 16 TB of data, the equivalent of list most data for dynamic acquisitions for roughly 25-30 patients using the Biograph Vision Quadra. It is therefore an understatement to state that the unprecedented volumes of data generated by a LAFOV system places undue burden on the IT networks available in most nuclear medicine centres. It also underscores the vital importance of interdisciplinary team-working when realising nuclear medicine research projects with LAFOV, where IT technicians, data scientists and nuclear medicine physicists have a vital role to play in helping to manage and manipulate these large datasets. Potential operators of these systems who foresee the need to store large amounts of raw data for research purposes will need to factor in the challenges in doing so when drawing up their plans. The concept of “big-data” has become cliché; if exciting ideas such as exploring the metabolic connectome using large numbers of PET datasets to explore organ-organ interactions are to be truly realised [38, 47], then the challenges inherent to storing, retrieving and sharing such large datasets using current technology and infrastructure need to be met.

There are a number of reports of low-activity protocols in conjunction with LAFOV scanners, often justified on grounds of dose reduction. Although there is a clear rationale to reducing radiation dose, the clinical benefit of doing so is not clear, as will be discussed later in this article. In our view, a more compelling justification, and one which is rarely mentioned in the low-dose literature, is the idea of data-economy: low-activity, low-count scans, potentially augmented by AI de-noising [48–50] are two potential lean-data solutions.

High volume, systems level analysis or artificial intelligence driven analysis of total-body PET data may reveal insights into multi-organ metabolic networks. However, the storage, retrieval and reconstruction of large volumes of raw data for research is clearly impracticable without dedicated approaches to storing, reconstructing and transferring data. It is correctly stated that a SAFOV system collects less than 1% of annihilation photons produced [26]. However, if these additional events detected by LAFOV cannot be marshalled and stored in a manageable fashion where list mode data can be retrieved, e.g. to subject them to further analysis such as radiomics, where study specific retrospective reconstruction of list mode data is required for harmonisation purposes [51, 52], or for the training of AI models directly on sinogramme data [53], the full panoply of benefits which the large datasets produced by LAFOV are at risk of being lost if mere static reconstructions of SUV maps are stored in clinical PACS systems for convenience.

### Assessing and implementing a disruptive technology

In an editorial in this journal, Hicks and van den Abbeele ask whether LAFOV will be an expensive folly or will become the next clinical standard for PET/CT [54]. We remain agnostic on this topic: while it is clear that total body imaging has a number of research applications which might inform future clinical practice, it remains to be seen whether the higher sensitivity offered by such scanners translates either to improved scan performance or are more cost effective compared to SAFOV systems. If LAFOV scanning is to become the next clinical standard, and not just restricted to blue-sky research at academic centres, high quality and prospective evidence is necessary. A search of clinicaltrials.gov for “total-body PET/CT” reveals nine prospective studies in various stages of completion, including one comparative imaging study at our own centre. As the number of centres operating LAFOV systems increases, we look forward to the publication of more studies which might help answer these questions.

In this regard, perhaps a salutary lesson can be learnt from the introduction of combined PET/MRI scanners. Although introduced with much fanfare as a “game-changing” development, after almost a decade, PET/MRI remains far from being adopted universally as a standard examination. Although the advantages of combining the high sensitivity of PET imaging with the high anatomical resolution of MRI are readily apparent [55], as is the possibility to reduce radiation exposure through omission of the CT component, there are no routine clinical indications where PET/MRI has become the standard of care, and a number of clinical and financial hurdles remain before PET/MRI can be adopted as a routine clinical examination [56, 57]. Consequently, PET/MRI has not replaced PET/CT in the majority of centres and remains as a complementary tool at a limited number of sites. The assessment of disruptive technologies can be challenging [58]. Traditional methods of evidence generation can take many years to obtain and the demand for evidence to justify expenditure can act as a hindrance to the adoption of cutting edge technologies. It is therefore an individual and centre-specific decision as to whether the potential benefits to installing and operating a LAFOV system justify the expenditure involved.

### From low dose to ultra-low dose

**Fig. 2:**
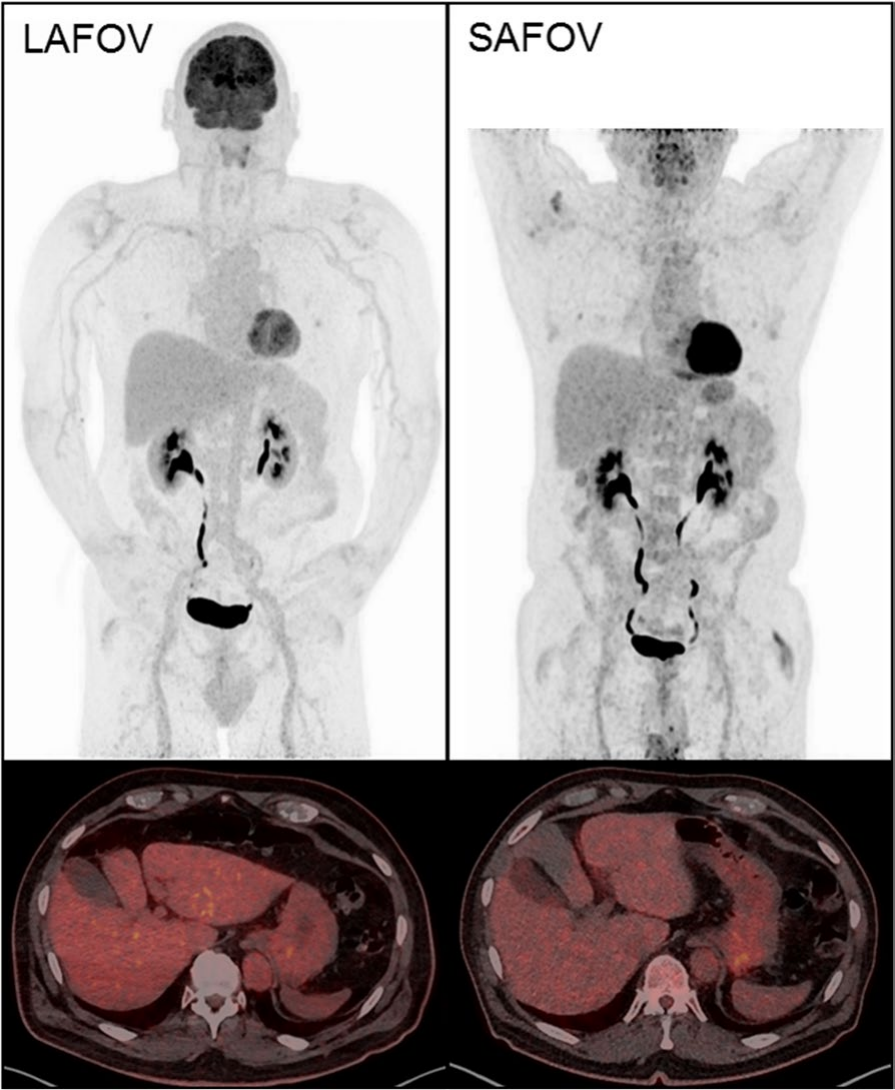
Indicative imaging quality for a low dose PET/CT acquisition on a LAFOV with 20 MBq of 2-[^18^F]FDG (left) and a full dose with 340 MBq on an analogue SAFOV system (right). The scan times for both are equivalent at 20 min. Maximal intensity projections (MIP) and co-registered PET/CT axial slices (bottom) for the same patient are shown

**Fig. 3:**
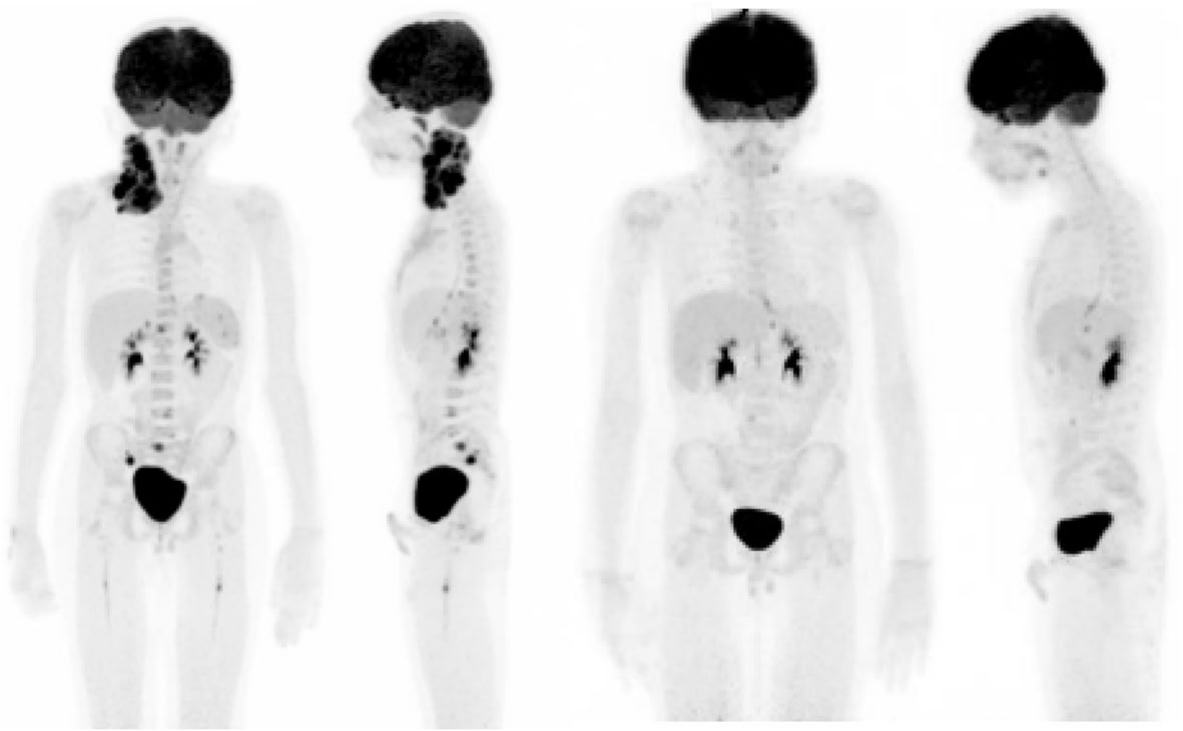
Indicative imaging quality for a low dose PET/CT acquisition in an 8 year old child (weight 27 kg) with Hodgkin Lymphoma. The images on the left were obtained in a 12 min scan using 27 MBq of 2-[^18^F]FDG, the images on the right were obtained in a 12 min acquisition using just 14 MBq of 2-[^18^F]FDG, resulting in an equivalent dose for the PET component of just 0.27 mSV

Instead of focussing on throughput, LAFOV systems can furnish substantial reductions in applied activity, and consequently the radiation dose to the patient and caregivers as well as allowing more patients to be examined for a given activity of radiopharmaceutical [26, 31]. In some applications, LAFOV systems can be used to scan where trace amounts of a radiotracer are available, for example when imaging long-half life radiopharmaceuticals such as [^89^Zr] or in low-count applications such as the imaging of [^90^Y] [59, 60]. In certain circumstances, very low activity acquisitions can be performed, such as the patient in Fig. 2 where 20.8 MBq was applied to an 86 kg adult male, and provided an image of good quality when compared to a previous scan on the same patient using an analogue scanner five years previously (Biograph mCT, Siemens Healthineers) with 340 MBq. A low dose approach lends itself very well to imaging children. In Fig. 3 we show an example of the type of image quality available with just 14 MBq of 2-[^18^F]FDG in an 8 year old child with Hodgkin Lymphoma. This results in an impressively low equivalent dose of 0.3 mSv for the PET component. Appropriateness criteria which justify alternative examinations based on equivalent dose alone in paediatric patients will need to be revisited [61]. Moreover, standard doses can be used but with shorter examination protocols, which might make the requirement for anaesthesia or sedation less likely, and PET/CT has potentially very important advantages in this regard compared to the lengthy scan procedures encountered in whole-body MRI [37]. Convention holds that PET/CT is a high equivalent dose modality, that ionising radiation in children is inherently bad and MRI with anaesthesia is post hoc *ergo propter hoc* safer [62]. The combination of fast anaesthesia free imaging [37, 63] with ionising radiation dose comparable to routine conventional radiographs might upend this received wisdom. Djekide et al. argue that with the advent of LAFOV systems, paediatric PET – historically underutilised [64, 65] is “ready for prime time” [61]. We agree with this sentiment entirely, and eagerly anticipate more studies assessing the potential benefits of paediatric LAFOV PET.

However, when reducing activity care must be taken to ensure that the scan quality is not compromised, where higher noise images can obscure small lesions [23, 66]. Dose optimisation is not synonymous with dose reduction, and activities should be defined according to their diagnostic yield rather than arbitrary or subjective image quality [67, 68]. The magnitude of any theoretical benefit to an individual patient through only modest reductions in radiation dose, for example a 50% reduction in activity [40] is questionable. Moreover, as nuclear medicine physicians, we must be careful that we do not inadvertently contribute to radiation induced phobia through overstating the risks of radiation doses routinely used in diagnostic procedures, which in many cases are smaller in magnitude than the risks inherent to the car journey to the hospital [69–72]. Furthermore, care must be taken that the potential advantages of a high-quality, low-noise examination are not unnecessarily forfeited in the pursuit of radiation doses lower than those already accepted as safe in routine clinical imaging. For example, the present generation of SiPM-based PET-scanners are known to demonstrate higher detection rates [15, 18–20], diagnostic certainty and inter-rater reliability [22] and these benefits could extend to LAFOV systems.

Concentrating efforts on applied activity also excludes one of the two hybrid modalities in PET/CT. Even when taking existing dose reduction techniques for the CT component [73] and protocol optimisation into account [74, 75], a low dose non-contrast enhanced CT for attenuation correction (AC) is still in the order of 1-3 millisieverts (mSv). Instead, novel approaches for AC are to exploit domain-integrated AI [76] or naturally occurring ^176^Lu in the scanner’s Lutetium Oxyorthosilicate scintillators. High sensitivity LAFOV systems are able to detect sufficient signal from these background radiation events and use them as a transmission source (LSO-TX) [77, 78]. The scanner can be configured to provide “CT-less” attenuation maps for the attenuation correction of PET scans, and can be performed either before or after the PET acquisition in a manner akin to the transmission source scans performed prior to the advent of PET/CT [79]. AI can also be used to estimate attenuation maps using non AC-PET images [78, 80]. When combined with low-activity scans, it is now possible to obtain quantitatively reliable PET images with radiation doses under 0.5 mSv. In contrast to a marginal improvement through 50% reduction in the PET activity only, this combined approach could take the modality from one of the most dose intensive modalities to being in the same bracket as plain radiographs [78, 81]. Discussions about radiation dose can be very emotive [67]; efforts to reduce them will very likely make PET a more viable modality in paediatrics and obstetric imaging [64, 82, 83], as a sensitive screening tool [84] and as a more acceptable tool for research with healthy individuals or for multiple-tracer studies. After nearly two decades of successful hybrid imaging [7] it might seem counterintuitive to use novel approaches to use CT-less attenuation techniques. After all, CT provides important anatomical detail and training programmes have been substantially reconfigured to ensure that nuclear medicine physicians are able to make maximum use of this information [9, 11–14, 85–87]. However, we envisage a number of situations where this approach might be useful. For example, when using a LAFOV scanner there are a number cases where whole-body PET data is incidentally acquired (for example whole body data for a dedicated brain acquisition). These data could then be attenuation corrected for review and analysis even though they do not lie in the clinical volume of interest. For example dedicated parathyroid and brain imaging, performing an additional diagnostic or AC-CT of the body is not clinically required or justified. In some circumstances, such as imaging of brain tumours, an anatomic modality such as MRI is already available, and the AC-CT has no additional diagnostic benefit. We can also envisage a role for CT-less reconstruction of data in research settings and for multiple-time point imaging.

### Rethinking imaging protocols

**Fig. 4:**
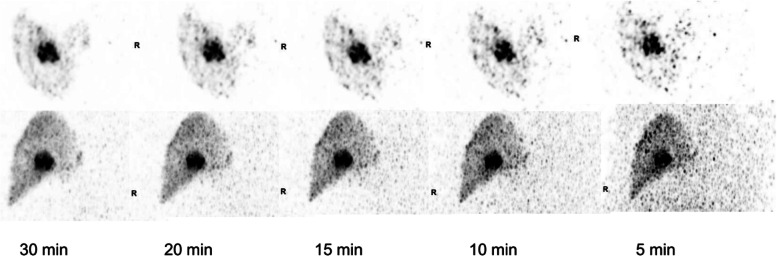
Example image quality following 1GBq of [^90^Y] Therasphere for treatment of hepatocellular carcinoma. [^90^Y] is traditionally challenging to image, either using Bremsstrahlung or as a result of the low (32 per million) rate of positron emission

**Fig. 5:**
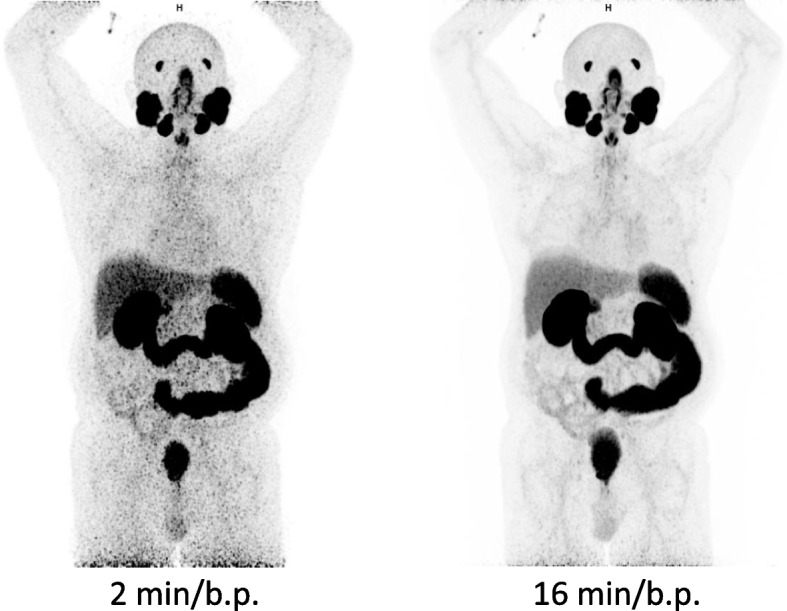
Example of a simulated “low-dose” or late acquisition at 4 h p.i. For comparison the 16 min/b.p. acquisition (right hand side) is shown alongside a sample re-binning of the same data for a guideline recommended 2 min/b.p. acquisition, with resultant reduction in image quality following a clinically standard application of 192 MBq of [^68^Ga]Ga-PSMA-11. Owing to the short half-life of [^68^Ga] (68 min) later acquisitions were limited by poor count statistics and noise. It should be noted that 2 min/bp in continuous bed motion (cbm) can take up to 16 min on previous generation SAFOV scanner designs

Free of the constraints imposed by lower sensitivity scanners, we have also had occasion to re-imagine a number of imaging protocols. For example, the recommended uptake time for [^68^Ga]Ga-PSMA-11 of 60–90 min post-injection (p.i.) is a compromise between applied activity and the short half-life of the radiotracer, where later imaging can result in intolerable image noise. However, this is not the optimal time-point for imaging from a pharmacokinetic perspective [88]. Increasing uptake over time increases lesion contrast. Later imaging might also be of diagnostic utility [89–91] and might provide improved prognostic information, for example in a preliminary work Abdelhafaz et al. demonstrated the superiority of Deauville scoring at 2 h compared to the standard 1 h using 2-[^18^F]FDG, and is another example of how imaging protocols which have been ingrained over many years might be revisited in the light of high sensitivity LAFOV systems [92]. Imaging can be possible over many half-lives without impairment in imaging quality [93] and impressive results have been obtained for very long half-life tracers such as [^89^Zr] [94]. Novel methods of imaging, such as using [^90^Y] post selective internal radiation therapy (SIRT) for liver malignancy is possible with impressive image quality using a tracer which was not traditionally considered amenable to PET (Fig. 4) [60]. From our own work, we have been able to demonstrate the advantage of imaging at later time-points when lesion uptake is higher and background clearance greater [89, 93, 95–97]. Previously, using lower sensitivity scanners, such systems were limited by the high image noise or the need to use substantially higher doses of the radiopharmaceutical. Now, using LAFOV systems, it is possible to obtain high count density acquisitions within reasonable time-frames: to achieve similar count statistics for a 10 min LAFOV acquisition with the Siemens Biograph Vision Quadra would take an impracticable 88 min using flow-motion with a table velocity of 0.2 mm/s. For example, in Fig. 5 we show the image quality achievable in a standard 2 min/bed position (for 106 cm FOV 16 min scan time) compared to a 16 min single bed position acquisition using a LAFOV system for a late acquisition at 4 h p.i, with [^68^Ga]Ga-PSMA-11. This higher sensitivity can be used to capture high quality images after nearly four half-lives, with improved lesion uptake and lower background. Moreover, this greater dynamic range captures a wider temporal range of kinetic data. The ability to perform multi-time point imaging is of great interest for theragnostic dosimetry [98]; it is currently a major shortcoming of radioligand therapy that individualised dose planning is, in many cases, not currently possible.

It is also clear that a number of tumour entities are best imaged by multiple tracers. For example, even with a state-of-the-art digital PET/CT and at high PSA values at recurrence, up to 5% of prostate cancers do not express sufficient the prostate specific membrane antigen (PSMA) to be usefully imaged by this technique [15]. Additional 2-[^18^F]FDG PET/CT is a useful adjunct for theragnostic assessment for PSMA-radioligand therapy, and combined FDG/PSMA PET might be useful in renal cell carcinoma [99]. As an alternative to multiple imaging sessions, or the need for two full-dose scans, the flexibility of the LAFOV to obtain qualitatively acceptable scans at low dose affords the possibility to seamlessly perform additional imaging with a second tracer at low-dose, or vice-versa [100]. One barrier to the implementation of dual-tracer protocols in PET/CT imaging is the inability to discriminate between the signal from each tracer, although the abbreviated dynamic protocols can be used to distinguish each tracer’s kinetic behaviour or the imaging of prompt gammas and triplets have been proposed to differentiate between a pure positron and non-pure positron emitters [101]; the high sensitivity of LAFOV lends itself to these methods well and is a feature which future users of these systems might explore [102].

## The future of PET is quantitative

**Fig. 6:**
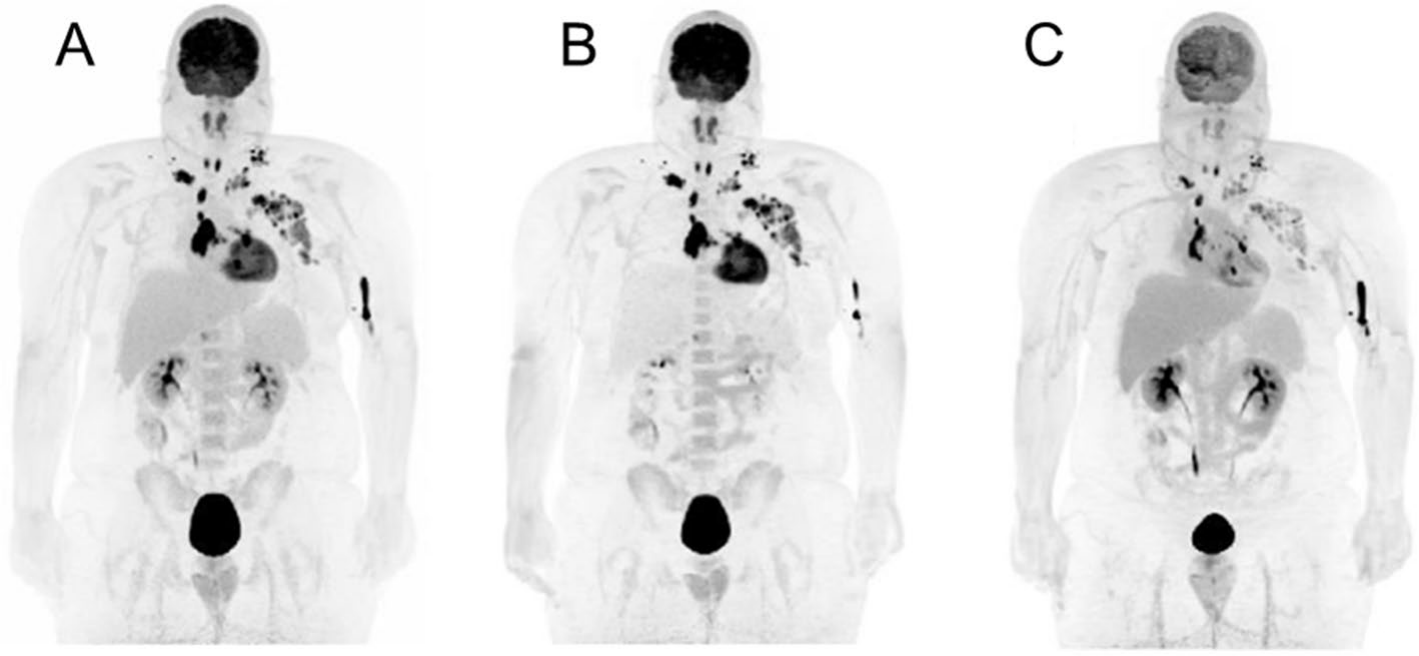
Example static SUV map for a patient with Lymphoma at 2-[^18^F]FDG PET/CT (**A**), Patlak K_i_ map demonstrating metabolically trapped FDG (**B**) and Patlak distribution volume (DV) showing non-trapped FDG (**C**)

While reduction in activity and assessment of subjective image quality is important, these represent only incremental improvements upon already established techniques. PET data are commonly presented by way of weight, activity and decay normalised uptake (SUV) maps which are semi-quantitative in nature and which physicians are trained to interpret in a qualitative and subjective fashion. The shortcomings of SUV [103] or the closely related metabolic tumour volume (MTV) are well known [104]. The few established or routinely reported scoring systems employ semi-quantitative, single-time point measurements of SUV relative to a reference organ or blood pool, such as the Deauville, Hopkins and Krenning scores. In contrast, there are established and quantitative methods for the analysis of dynamic PET data which can reveal deeper insights into physiological parameters that are not possible from single-time point analysis, such as the Patlak-Gjeddes plot [105]. Despite clear benefits to this approach [39], such as improved lesion detection though higher tumour to background ratios [44], they have enjoyed very limited adoption in routine clinical imaging. Four decades on from Patlak’s pioneering work, nuclear medicine images are still presented in semi-quantitative SUV maps and there are no clinically validated uses for kinetic modelling in diagnostic imaging. There are a number of reasons why this is the case. In our view the requirement for a patient to be scanned for up to an hour (e.g. in a dynamic 2-[^18^F]FDG PET scan) is a clear barrier in an era where PET scans can be obtained within minutes (although it should be noted that similar scan times are routinely encountered in whole body MRI, and the increased scan capacity generated by reduced acquisition times could be used flexibly to accommodate this in selected patients). However, LAFOV scanners with higher sensitivity open up the possibility for quantitative imaging techniques to be translated into the clinic with comparable time frames to static acquisition such as an abbreviated 20 min protocol, aided by dual-phase, combined population-based input function protocols or AI approaches [42, 106–111]. Noise in dynamic acquisitions directly contributes to noise in kinetic parameter estimates; for the first time LAFOV systems allow a radiopharmaceutical to be traced throughout the entire body with excellent temporal and spatial resolution, as shown in Fig. 1. Previously, the requirement for an input function restricted SAFOV analysis to a single organ or region. Many studies must first be performed before abbreviated dynamic imaging can become a validated and routine clinical tool rather than as a research tool. Nevertheless, LAFOV makes it feasible to perform whole-body quantitative kinetic analysis within currently routine scanning times. An example patient with whole-body kinetic analysis performed on a LAFOV is shown in Fig. 6. As shown in this figure, high quality Patlak tracer flux (Ki) images have improved target-to-background compared to the SUV images. Furthermore, the distribution volume (DV) images can provide additional biological information regarding the Lymphoma. Previously, the complexity of parametric imaging analysis made this technique available only to centres with advanced tools and kinetic imaging experts. Now, automated software which can perform direct parametric imaging analysis is available on some commercial PET scanners and extension of this software to LAFOV will allow the design of more effective clinical protocols for practical clinical parametric imaging without the need for additional complex or time-consuming image analysis.

### Training the molecular imager of the future

Cognisant of a preponderance of data that fully quantitative kinetic analysis will yield superior results to traditional single-time point imaging, if successfully translated into clinical routine, it promises to fundamentally alter the way in which PET data are interpreted and reported. In recent decades, training programmes in nuclear medicine have been reconfigured with heavy emphasis on training in anatomic imaging modalities or the training of dual-certified radionuclide radiologists. Consequently, in many countries there has been a decline in the number of nuclear medicine physicians being trained [85–87]. We predict that LAFOV PET could act as a catalyst for truly quantitative functional and kinetic imaging. With exciting possibilities on the horizon, such as personalised cocktails of multiple tracers or the ability to probe whole-body metabolic connectome data, interesting questions will arise about how we might best train and educate nuclear medicine residents to best exploit these technologies. Whereas physician training has previously emphasised mastery of anatomical imaging modalities, nuclear-medicine specific skills and knowledge, such as the ability to code and manage data, perform and interpret kinetic modelling and knowledge of advanced radiomics techniques might need to be given greater weight to educate the molecular imager and therapist of the future.

### Low dose or low noise?

LAFOV PET images are characterised by their high temporal and spatial resolution. Cinematic rendering provides life-like 3D visualisation of anatomical structures which can be useful in educational scenarios and in demonstrating complex 3D anatomy in multidisciplinary meetings, and can now be applied to PET-data also [112]. Moreover, we posit that these low-noise and high signal images can afford improvements in PET quantification and textural analysis: indeed radiomics is based upon the premise that additional biological information is contained in these features [113–115]. Despite claims made for its superiority over traditional methods of PET image interpretation and decades of research effort, there is still no routine clinical application for radiomics analysis and only limited consensus about how such images are best reconstructed, analysed and interpreted. There has been a surfeit of studies which cannot be replicated [116, 117] and numerous features are redundant and non-reproducible [118]. Amongst the numerous issues to be addressed in obtaining repeatable and reliable radiomics data [51, 119–122], a particular problem is their sensitivity to noise [123], for which low-noise high-quality LAFOV acquisitions might be well positioned to address.

### Future directions in LAFOV scanning

The pioneering PennPET and the first commercially available systems (uExplorer and Siemens Biograph Vision Quadra) are certainly not going to be the last word on LAFOV PET and further developments in PET technology are eagerly anticipated. For example, where the cost of the scanner is tightly linked to the cost of the scintillation crystals, sparse detector designs are a potential solution to improvement in axial coverage without increase in scanner cost [124], although this will come at the price of lower sensitivity. The use of cheaper BGO crystals is a possibility, but more clarity is currently required about the performance of such systems, such as TOF capability and count rate performance.

Any efforts to reduce the capital cost associated with PET/CT is welcome; this resource intensive imaging modality is not just the luxury of rich economies, but represents a standard of care for a wide variety of indications and is increasingly performed in low and middle income countries, where inequality in access is an important issue [125]. An exciting design for an entirely novel PET/CT system is the Jagiellonian PET (J-PET). The use of plastic scintillation detectors has multiple advantages in terms of engineering practicability, cost, weight and sensitivity. They are compatible for MRI inserts and exhibit substantially faster scintillation time (0.5 ns versus 40 ns for LSO and 300 ns for BGO). This ultrafast time-of-flight (TOF) resolution could also provide direct knowledge of the annihilation location and might even obviate the need for reconstruction algorithms [126, 127]. Although much work is required before such systems can be introduced into clinical operation, these exciting developments demonstrate that hardware development for PET/CT is far from over. Needless to say, these systems might also compound the aforementioned data storage issues; in our view this gives further cause for urgency in resolving these issues.

Whereas traditional scintillation crystals have registered the incident photon via the photoelectric effect, the plastic scintillators can determine the polarisation of the annihilation photon through the detection of primary and secondary Compton scattering. Intriguingly, this raises the notion of extracting quantum information from metabolic processes in the body [128, 129], e.g. providing a non-invasive and quantitative means to interrogating tissue hypoxia in vivo [130–134]. Moreover, beyond sensitivity and TOF-resolution, PET-data is limited by its relatively poor spatial resolution compared to CT or MRI [5]. Future scanner designs may address this limitation, e.g. it is possible that ultra-fast TOF resolution might also aid spatial resolution [135, 136].

## Conclusion

There have been many important developments in PET technology over its long history. However, at several points technological developments have resulted in sea changes for the field, most notably the introduction of hybrid imaging which led to the rapid incorporation of PET/CT for the standard investigation of a plethora of diseases and had significant implications for the training and organisation of our field. Although LAFOV systems allow room for some reduction in applied activity or faster acquisitions, we argue that these represent only the low-hanging fruits amongst the myriad of benefits that this new technology can offer. Instead, we are convinced that the sum of a LAFOV system is greater than the total of its parts: we are particularly encouraged by the ability to obtain whole-body tracer kinetics, ultra-low noise and high count rate data which, for the first time, makes the adoption of quantitative kinetic analysis feasible and promises to reveal deeper insights into human pathophysiology and function which traditional, single time-point and semi-quantitative analysis of PET data cannot provide. The increased sensitivity allows a greater dynamic range, meaning that tracers can be imaged over many more half-lives capturing a wider range of biokinetics, or multiple time point imaging which could be of assistance in dosimetry. Finally, the ability to combine two or more low-dose examinations makes a more comprehensive interrogation of tumour-biology possible with multi-tracer protocols. Although in their infancy, innovative solutions to improve sensitivity through novel detector materials and faster TOF may fundamentally change the way PET data is obtained, reconstructed and interpreted.

## Data Availability

Not applicable.

## Abbreviations

2-[18F]-FDG: 2-deoxy-2-[18F]fluoro-D-glucose
aFOV: Axial field-of-view
BGO: Bismuth Germanate
CBM: Continuous bed motion
CT: Computed tomography
FOV: Field-of-view
LAFOV: Long-axial field-of-view
MRI: Magnetic resonance imaging
PACS: Picture archival and communication system
PET/CT: Positron emission and computed tomographies
PMT: Photon multiplier tube
SAFOV: Standard-axial field-of-view
SiPM: Silicon Photomultiplier
SIRT: Selective internal radiation therapy
SUV: Standardised uptake value
TOF: Time-of-flight

## References

[1] Sweet WH (1951). The uses of nuclear disintegration in the diagnosis and treatment of brain tumor. N Engl J Med.

[2] Wrenn FR, Good ML, Handler P (1951). The use of positron-emitting radioisotopes for the localization of brain tumors. Science.

[3] Kuhl DE, Phelps ME, Hoffman EJ, Robinson GD, MacDonald NS (1977). Initial clinical experience with 18F-2-fluoro-2-deoxy-d-glucose for determination of local cerebral glucose utilization by emission computed tomography. Acta Neurol Scand Suppl.

[4] Petroni D, Menichetti L, Poli M (2020). Historical and radiopharmaceutical relevance of [18F]FDG. J Radioanal Nucl Chem.

[5] Budinger TF (1998). PET instrumentation: what are the limits?. Semin Nucl Med.

[6] Ter-Pogossian MM, Phelps ME, Hoffman EJ, Mullani NA (1975). A positron-emission transaxial tomograph for nuclear imaging (PETT). Radiology.

[7] Beyer T, Townsend DW, Brun T, Kinahan PE, Charron M, Roddy R (2000). A combined PET/CT scanner for clinical oncology. J Nucl Med.

[8] Virgolini I, Decristoforo C, Haug A, Fanti S, Uprimny C (2018). Current status of theranostics in prostate cancer. Eur J Nucl Med Mol Imaging.

[9] Czernin J, Sonni I, Razmaria A, Calais J (2019). The future of nuclear medicine as an independent specialty. J Nucl Med.

[10] Delbeke D, Segall GM (2011). Status of and trends in nuclear medicine in the United States. J Nucl Med.

[11] Mankoff D, Pryma DA (2017). Nuclear medicine training: what now?. J Nucl Med.

[12] Muylle K, Maffioli L (2017). Nuclear medicine training in Europe: “all for one, one for all”. J Nucl Med.

[13] Neilly B, Dizdarevic S, Prvulovich L, Buscombe J, Lewington V (2016). Nuclear medicine training and practice in the UK. Eur J Nucl Med Mol Imaging.

[14] Segall GM, Grady EE, Fair JR, Ghesani MV, Gordon L (2017). Nuclear medicine training in the United States. J Nucl Med.

[15] Alberts I, Prenosil G, Sachpekidis C, Weitzel T, Shi K, Rominger A (2020). Digital versus analogue PET in [(68)Ga]Ga-PSMA-11 PET/CT for recurrent prostate cancer: a matched-pair comparison. Eur J Nucl Med Mol Imaging.

[16] Rausch I, Ruiz A, Valverde-Pascual I, Cal-Gonzalez J, Beyer T, Carrio I (2019). Performance evaluation of the Vereos PET/CT system according to the NEMA NU2-2012 standard. J Nucl Med.

[17] van Sluis JJ, de Jong J, Schaar J, Noordzij W, van Snick P, Dierckx R (2019). Performance characteristics of the digital biograph vision PET/CT system. J Nucl Med.

[18] Nguyen NC, Vercher-Conejero JL, Sattar A, Miller MA, Maniawski PJ, Jordan DW (2015). Image quality and diagnostic performance of a digital PET prototype in patients with oncologic diseases: initial experience and comparison with analog PET. J Nucl Med.

[19] Fuentes-Ocampo F, Lopez-Mora DA, Flotats A, Paillahueque G, Camacho V, Duch J (2019). Digital vs. analog PET/CT: intra-subject comparison of the SUVmax in target lesions and reference regions. Eur J Nucl Med Mol Imaging.

[20] Lopez-Mora DA, Flotats A, Fuentes-Ocampo F, Camacho V, Fernandez A, Ruiz A (2019). Comparison of image quality and lesion detection between digital and analog PET/CT. Eur J Nucl Med Mol Imaging.

[21] van Sluis J, Boellaard R, Dierckx RA, Stormezand G, Glaudemans A, Noordzij W. Image quality and activity optimization in oncological (18)F-FDG PET using the digital biograph vision PET/CT. J Nucl Med. 2019. 10.2967/jnumed.119.234351. 10.2967/jnumed.119.234351 31628214

[22] Alberts I, Hünermund J-N, Sachpekidis C, Mingels C, Fech V, Bohn KP, et al. The influence of digital PET/CT on diagnostic certainty and interrater reliability in [68Ga]Ga-PSMA-11 PET/CT for recurrent prostate cancer. Eur Radiol. 2021. 10.1007/s00330-021-07870-5. 10.1007/s00330-021-07870-5 PMC8452558 33856522

[23] Alberts I, Sachpekidis C, Prenosil G, Viscione M, Bohn KP, Mingels C (2021). Digital PET/CT allows for shorter acquisition protocols or reduced radiopharmaceutical dose in [18F]-FDG PET/CT. Ann Nucl Med.

[24] Zeimpekis KG, Kotasidis FA, Huellner M, Nemirovsky A, Kaufmann PA, Treyer V. NEMA NU 2-2018 performance evaluation of a new generation 30-cm axial field-of-view discovery MI PET/CT. Eur J Nucl Med Mol Imaging. 2022. 10.1007/s00259-022-05751-7. 10.1007/s00259-022-05751-7 PMC9250480 35284970

[25] Daube-Witherspoon ME, Cherry SR (2021). Scanner design considerations for long axial field-of-view PET systems. PET Clin.

[26] Cherry SR, Jones T, Karp JS, Qi J, Moses WW, Badawi RD (2018). Total-body PET: maximizing sensitivity to create new opportunities for clinical research and patient care. J Nucl Med.

[27] Karp JS, Viswanath V, Geagan MJ, Muehllehner G, Pantel AR, Parma MJ (2020). PennPET Explorer: design and preliminary performance of a whole-body imager. J Nucl Med.

[28] Pantel AR, Viswanath V, Karp JS (2021). Update on the PennPET Explorer: a whole-body imager with scalable axial field-of-view. PET Clin.

[29] Daube-Witherspoon M, Pantel A, Pryma D, Karp J. Total-body PET: a new paradigm for molecular imaging. Br J Radiol. 2022:20220357. 10.1259/bjr.20220357. 10.1259/bjr.20220357 PMC9733603 35993615

[30] Lan X, Younis MH, Li K, Cai W (2021). First clinical experience of 106 cm, long axial field-of-view (LAFOV) PET/CT: an elegant balance between standard axial (23 cm) and total-body (194 cm) systems. Eur J Nucl Med Mol Imaging.

[31] Alberts I, Hünermund J-N, Prenosil G, Mingels C, Bohn KP, Viscione M, et al. Clinical performance of long axial field of view PET/CT: a head-to-head intra-individual comparison of the Biograph Vision Quadra with the Biograph Vision PET/CT. Eur J Nucl Med Mol Imaging. 2021. 10.1007/s00259-021-05282-7. 10.1007/s00259-021-05282-7 PMC8241747 33797596

[32] Prenosil GA, Sari H, Furstner M, Afshar-Oromieh A, Shi K, Rominger A (2022). Performance characteristics of the Biograph Vision Quadra PET/CT system with a long axial field of view using the NEMA NU 2-2018 standard. J Nucl Med.

[33] Gourd K, Collingridge D (2021). Improving the view: the need for global action on universal access to cancer imaging. Lancet Oncol.

[34] Anderson JA (2005). TH-A-I-617-01: PET site planning and radiation safety. Med Phys.

[35] Sachpekidis C, Pan L, Kopp-Schneider A, Weru V, Hassel JC, Dimitrakopoulou-Strauss A. Application of the long axial field-of-view PET/CT with low-dose [(18)F]FDG in melanoma. Eur J Nucl Med Mol Imaging. 2022. 10.1007/s00259-022-06070-7. 10.1007/s00259-022-06070-7 PMC9931831 36474125

[36] Vali R, Alessio A, Balza R, Borgwardt L, Bar-Sever Z, Czachowski M (2021). SNMMI procedure standard/EANM practice guideline on pediatric (18)F-FDG PET/CT for oncology 1.0. J Nucl Med.

[37] van Rijsewijk ND, van Leer B, Ivashchenko OV, Scholvinck EH, van den Heuvel F, van Snick JH, et al. Ultra-low dose infection imaging of a newborn without sedation using long axial field-of-view PET/CT. Eur J Nucl Med Mol Imaging. 2022. 10.1007/s00259-022-05979-3. 10.1007/s00259-022-05979-3 PMC9816243 36166078

[38] Shiyam Sundar LK, Hacker M, Beyer T. Whole-body PET imaging: a catalyst for whole-person research? J Nucl Med. 2022. 10.2967/jnumed.122.264555. 10.2967/jnumed.122.264555 PMC9902855 36460342

[39] Rahmim A, Lodge MA, Karakatsanis NA, Panin VY, Zhou Y, McMillan A (2019). Dynamic whole-body PET imaging: principles, potentials and applications. Eur J Nucl Med Mol Imaging.

[40] Tan H, Sui X, Yin H, Yu H, Gu Y, Chen S (2021). Total-body PET/CT using half-dose FDG and compared with conventional PET/CT using full-dose FDG in lung cancer. Eur J Nucl Med Mol Imaging.

[41] Duarte PS (2021). Give to Fryback what is Fryback’s, and to new PET technologies what is new PET technologies’. Eur J Nucl Med Mol Imaging.

[42] Sari H, Mingels C, Alberts I, Hu J, Buesser D, Shah V, et al. First results on kinetic modelling and parametric imaging of dynamic (18)F-FDG datasets from a long axial FOV PET scanner in oncological patients. Eur J Nucl Med Mol Imaging. 2022. 10.1007/s00259-021-05623-6. 10.1007/s00259-021-05623-6 34981164

[43] Dimitrakopoulou-Strauss A, Pan L, Sachpekidis C (2021). Kinetic modeling and parametric imaging with dynamic PET for oncological applications: general considerations, current clinical applications, and future perspectives. Eur J Nucl Med Mol Imaging.

[44] Fahrni G, Karakatsanis NA, Di Domenicantonio G, Garibotto V, Zaidi H (2019). Does whole-body Patlak (18)F-FDG PET imaging improve lesion detectability in clinical oncology?. Eur Radiol.

[45] Katal S, Eibschutz LS, Saboury B, Gholamrezanezhad A, Alavi A. Advantages and applications of total-body PET scanning. Diagnostics (Basel). 2022;12. 10.3390/diagnostics12020426. 10.3390/diagnostics12020426 PMC8871405 35204517

[46] Alavi A, Saboury B, Nardo L, Zhang V, Wang M, Li H (2022). Potential and most relevant applications of total body PET/CT imaging. Clin Nucl Med.

[47] Sun T, Wang Z, Wu Y, Gu F, Li X, Bai Y (2022). Identifying the individual metabolic abnormities from a systemic perspective using whole-body PET imaging. Eur J Nucl Med Mol Imaging.

[48] Cui J, Gong K, Guo N, Wu C, Meng X, Kim K (2019). PET image denoising using unsupervised deep learning. Eur J Nucl Med Mol Imaging.

[49] Gong K, Guan J, Liu CC, Qi J (2019). PET image denoising using a deep neural network through fine tuning. IEEE Trans Radiat Plasma Med Sci.

[50] Xue S, Guo R, Bohn KP, Matzke J, Viscione M, Alberts I (2022). A cross-scanner and cross-tracer deep learning method for the recovery of standard-dose imaging quality from low-dose PET. Eur J Nucl Med Mol Imaging.

[51] Orlhac F, Eertink JJ, Cottereau AS, Zijlstra JM, Thieblemont C, Meignan M (2022). A guide to ComBat harmonization of imaging biomarkers in multicenter studies. J Nucl Med.

[52] Da-Ano R, Lucia F, Masson I, Abgral R, Alfieri J, Rousseau C (2021). A transfer learning approach to facilitate ComBat-based harmonization of multicentre radiomic features in new datasets. PLoS One.

[53] Ma R, Hu J, Sari H, Xue S, Mingels C, Viscione M, et al. An encoder-decoder network for direct image reconstruction on sinograms of a long axial field of view PET. Eur J Nucl Med Mol Imaging. 2022. 10.1007/s00259-022-05861-2. 10.1007/s00259-022-05861-2 35819497

[54] Hicks RJ, Van den Abbeele AD (2022). Will ultra-extended field-of-view scanners be an expensive folly or the next clinical standard for PET/CT?. Cancer Imaging.

[55] Antoch G, Bockisch A (2009). Combined PET/MRI: a new dimension in whole-body oncology imaging?. Eur J Nucl Med Mol Imaging.

[56] Spick C, Herrmann K, Czernin J (2016). 18F-FDG PET/CT and PET/MRI perform equally well in cancer: evidence from studies on more than 2,300 patients. J Nucl Med.

[57] Beyer T, Hacker M, Goh V (2017). PET/MRI-knocking on the doors of the rich and famous. Br J Radiol.

[58] Sounderajah V, Patel V, Varatharajan L, Harling L, Normahani P, Symons J (2021). Are disruptive innovations recognised in the healthcare literature? A systematic review. BMJ Innov.

[59] Brouwers AH, van Sluis J, van Snick JH, Schroder CP, Baas IO, Boellaard R, et al. First-time imaging of [(89)Zr]trastuzumab in breast cancer using a long axial field-of-view PET/CT scanner. Eur J Nucl Med Mol Imaging. 2022. 10.1007/s00259-022-05777-x. 10.1007/s00259-022-05777-x PMC9308603 35362794

[60] Zeimpekis KG, Mercolli L, Conti M, Sari H, Prenosil G, Shi K, et al. Phantom-based evaluation of yttrium-90 datasets using biograph vision quadra. Eur J Nucl Med Mol Imaging. 2022. 10.1007/s00259-022-06074-3. 10.1007/s00259-022-06074-3 PMC9931793 36504278

[61] Djekidel M, AlSadi R, Akl MA, Vandenberghe S, Bouhali O (2022). Total-body pediatric PET is ready for prime time. Eur J Nucl Med Mol Imaging.

[62] Callahan MJ, MacDougall RD, Bixby SD, Voss SD, Robertson RL, Cravero JP (2018). Ionizing radiation from computed tomography versus anesthesia for magnetic resonance imaging in infants and children: patient safety considerations. Pediatr Radiol.

[63] Reichkendler M, Andersen FL, Borgwardt L, Nygaard U, Albrecht-Beste E, Andersen KF, et al. Long axial field of view with 5 min acquisition time enables PET/CT in toddler without sedation. J Nucl Med. 2022:jnumed.121.263626. 10.2967/jnumed.121.263626. 10.2967/jnumed.121.263626 35710737

[64] Roca I, Simo M, Sabado C, de Toledo JS (2007). PET/CT in paediatrics: it is time to increase its use!. Eur J Nucl Med Mol Imaging.

[65] Hahn K, Pfluger T (2006). Is PET/CT necessary in paediatric oncology?. Eur J Nucl Med Mol Imaging.

[66] Rauscher I, Fendler WP, Hope TA, Quon A, Nekolla SG, Calais J (2020). Can the injected dose be reduced in (68)Ga-PSMA-11 PET/CT while maintaining high image quality for lesion detection?. J Nucl Med.

[67] McCready VR, Dizdarevic S (2018). Nuclear medicine RIP (radiation induced phobia); improving the image. Eur J Nucl Med Mol Imaging.

[68] McCready VR, Dizdarevic S, Beyer T (2020). Lesion detection and administered activity. J Nucl Med.

[69] Oakley PA, Harrison DE (2021). Are continued efforts to reduce radiation exposures from X-rays warranted?. Dose Response.

[70] Hall EJ, Brenner DJ (2012). Cancer risks from diagnostic radiology: the impact of new epidemiological data. Br J Radiol.

[71] Hall EJ, Brenner DJ (2008). Cancer risks from diagnostic radiology. Br J Radiol.

[72] Hendrick RE (2010). Radiation doses and cancer risks from breast imaging studies. Radiology.

[73] Greess H, Nomayr A, Wolf H, Baum U, Lell M, Bowing B (2002). Dose reduction in CT examination of children by an attenuation-based on-line modulation of tube current (CARE Dose). Eur Radiol.

[74] Gould SM, Mackewn J, Chicklore S, Cook GJR, Mallia A, Pike L (2021). Optimisation of CT protocols in PET-CT across different scanner models using different automatic exposure control methods and iterative reconstruction algorithms. EJNMMI Phys.

[75] Harun HH, Karim MKA, Abbas Z, Sabarudin A, Muniandy SC, Razak HRA (2021). The influence of iterative reconstruction level on image quality and radiation dose in CT pulmonary angiography examinations. Radiat Phys Chem.

[76] Guo R, Xue S, Hu J, Sari H, Mingels C, Zeimpekis K, et al. Using domain knowledge for robust and generalizable deep learning-based CT-free PET attenuation and scatter correction. Nat Commun. 2022; in press. 10.1038/s41467-022-33562-9 PMC9537165 36202816

[77] Teimoorisichani M, Panin V, Rothfuss H, Sari H, Rominger A, Conti M (2022). A CT-less approach to quantitative PET imaging using the LSO intrinsic radiation for long-axial FOV PET scanners. Med Phys.

[78] Teimoorisichani M, Sari H, Panin V, Bharkhada D, Rominger A, Conti M (2021). Using LSO background radiation for CT-less attenuation correction of PET data in long axial FOV PET scanners. J Nucl Med.

[79] Karp JS, Muehllehner G, Qu H, Yan XH (1995). Singles transmission in volume-imaging PET with a 137Cs source. Phys Med Biol.

[80] Xue S, Karl Peter B, Guo R, Sari H, Viscione M, Rominger A (2021). Development of a deep learning method for CT-free attenuation correction for a long axial field of view PET scanner. J Nucl Med.

[81] Sari H, Teimoorisichani M, Mingels C, Alberts I, Panin V, Bharkhada D, et al. Quantitative evaluation of a deep learning-based framework to generate whole-body attenuation maps using LSO background radiation in long axial FOV PET scanners. Eur J Nucl Med Mol Imaging. 2022. 10.1007/s00259-022-05909-3. 10.1007/s00259-022-05909-3 PMC9606046 35852557

[82] Korsholm K, Aleksyniene R, Albrecht-Beste E, Vadstrup ES, Andersen FL, Fischer BM. Staging of breast cancer in pregnancy with ultralow dose [18F]-FDG-PET/CT. Eur J Nucl Med Mol Imaging. 2022. 10.1007/s00259-022-06076-1. 10.1007/s00259-022-06076-1 36508027

[83] Zanotti-Fregonara P (2012). Pregnancy should not rule out 18FDG PET/CT for women with cancer. Lancet.

[84] Schöder H, Gönen M (2007). Screening for cancer with PET and PET/CT: potential and limitations. J Nucl Med.

[85] Velleman T, Kwee TC, Dierckx R, Ongena YP, Noordzij W. The integrated nuclear medicine and radiology residency program in the Netherlands: strengths and potential areas for improvement according to nuclear medicine physicians and radiologists. Eur J Nucl Med Mol Imaging. 2022. 10.1007/s00259-022-05699-8. 10.1007/s00259-022-05699-8 PMC9250465 35194672

[86] Velleman T, Noordzij W, Dierckx R, Ongena Y, Kwee TC (2021). The new integrated nuclear medicine and radiology residency program in the Netherlands: why do residents choose to subspecialize in nuclear medicine and why not?. J Nucl Med.

[87] Harolds JA, Metter D, Oates ME, Guiberteau MJ (2015). CT training of nuclear medicine residents in the United States, 2013-2014. J Am Coll Radiol.

[88] Afshar-Oromieh A, Hetzheim H, Kubler W, Kratochwil C, Giesel FL, Hope TA (2016). Radiation dosimetry of (68)Ga-PSMA-11 (HBED-CC) and preliminary evaluation of optimal imaging timing. Eur J Nucl Med Mol Imaging.

[89] Alberts I, Sachpekidis C, Dijkstra L, Prenosil G, Gourni E, Boxler S (2020). The role of additional late PSMA-ligand PET/CT in the differentiation between lymph node metastases and ganglia. Eur J Nucl Med Mol Imaging.

[90] Alberts I, Sachpekidis C, Gourni E, Boxler S, Gross T, Thalmann G (2020). Dynamic patterns of [(68)Ga]Ga-PSMA-11 uptake in recurrent prostate cancer lesions. Eur J Nucl Med Mol Imaging.

[91] Hustinx R, Smith RJ, Benard F, Rosenthal DI, Machtay M, Farber LA (1999). Dual time point fluorine-18 fluorodeoxyglucose positron emission tomography: a potential method to differentiate malignancy from inflammation and normal tissue in the head and neck. Eur J Nucl Med.

[92] Abdelhafez Y, Sen F, Tuscano J, Stephen M, Spencer B, Cherry S (2021). Differences in Deauville scores generated using 60- and 120-minute uptake times on total-body 18F-FDG PET/CT scans. J Nucl Med.

[93] Alberts I, Prenosil G, Mingels C, Bohn KP, Viscione M, Sari H (2021). Feasibility of late acquisition [68Ga]Ga-PSMA-11 PET/CT using a long axial field-of-view PET/CT scanner for the diagnosis of recurrent prostate cancer-first clinical experiences. Eur J Nucl Med Mol Imaging.

[94] Beckford Vera D, Schulte B, Henrich T, Flavell R, Seo Y, Abdelhafez Y (2020). First-in-human total-body PET imaging of HIV with 89Zr-VRC01 on the EXPLORER. J Nucl Med.

[95] Alberts I, Huenermund JN, Sachpekidis C, Zacho HD, Mingels C, Dijkstra L, et al. Combination of forced diuresis with additional late imaging in 68Ga-PSMA-11 PET/CT – effects on lesion visibility and radiotracer uptake. J Nucl Med. 2021:jnumed.120.257741. 10.2967/jnumed.120.257741. 10.2967/jnumed.120.257741 33547214

[96] Hoffmann MA, Buchholz HG, Wieler HJ, Rosar F, Miederer M, Fischer N, et al. Dual-time point [(68)Ga]Ga-PSMA-11 PET/CT hybrid imaging for staging and restaging of prostate cancer. Cancers (Basel). 2020;12. 10.3390/cancers12102788. 10.3390/cancers12102788 PMC7600341 32998432

[97] Afshar-Oromieh A, Hetzheim H, Kratochwil C, Benesova M, Eder M, Neels OC (2015). The theranostic PSMA ligand PSMA-617 in the diagnosis of prostate cancer by PET/CT: biodistribution in humans, radiation dosimetry, and first evaluation of tumor lesions. J Nucl Med.

[98] Ng QK-T, Triumbari EKA, Omidvari N, Cherry SR, Badawi RD, Nardo L (2022). Total-body PET/CT – first clinical experiences and future perspectives. Semin Nucl Med.

[99] Tariq A, Kwok M, Pearce A, Rhee H, Kyle S, Marsh P (2022). The role of dual tracer PSMA and FDG PET/CT in renal cell carcinoma (RCC) compared to conventional imaging: a multi-institutional case series with intra-individual comparison. Urol Oncol.

[100] Alberts I, Schepers R, Zeimpekis K, Sari H, Rominger A, Afshar-Oromieh A. Combined [68 Ga]Ga-PSMA-11 and low-dose 2-[18F]FDG PET/CT using a long-axial field of view scanner for patients referred for [177Lu]-PSMA-radioligand therapy. Eur J Nucl Med Mol Imaging. 2022. 10.1007/s00259-022-05961-z. 10.1007/s00259-022-05961-z PMC9852199 36136102

[101] Conti M, Eriksson L (2016). Physics of pure and non-pure positron emitters for PET: a review and a discussion. EJNMMI Phys.

[102] Abuelhia E, Kacperski K, Kafala S, Spyrou NM (2007). Performance of triple coincidence imaging as an addition to dedicated PET. Radiat Phys Chem.

[103] Keyes JW (1995). SUV: standard uptake or silly useless value?. J Nucl Med.

[104] Mikhaeel NG, Heymans MW, Eertink JJ, de Vet HCW, Boellaard R, Duhrsen U (2022). Proposed new dynamic prognostic index for diffuse large B-cell lymphoma: international metabolic prognostic index. J Clin Oncol.

[105] Patlak CS, Blasberg RG, Fenstermacher JD (1983). Graphical evaluation of blood-to-brain transfer constants from multiple-time uptake data. J Cereb Blood Flow Metab.

[106] Liu G, Yu H, Shi D, Hu P, Hu Y, Tan H (2022). Short-time total-body dynamic PET imaging performance in quantifying the kinetic metrics of (18)F-FDG in healthy volunteers. Eur J Nucl Med Mol Imaging.

[107] Viswanath V, Sari H, Pantel AR, Conti M, Daube-Witherspoon ME, Mingels C, et al. Abbreviated scan protocols to capture (18)F-FDG kinetics for long axial FOV PET scanners. Eur J Nucl Med Mol Imaging. 2022. 10.1007/s00259-022-05747-3. 10.1007/s00259-022-05747-3 PMC10695012 35278108

[108] Zhang X, Xie Z, Berg E, Judenhofer MS, Liu W, Xu T (2020). Total-body dynamic reconstruction and parametric imaging on the uEXPLORER. J Nucl Med.

[109] van Sluis J, Yaqub M, Brouwers AH, Dierckx RAJO, Noordzij W, Boellaard R (2021). Use of population input functions for reduced scan duration whole-body Patlak 18F-FDG PET imaging. EJNMMI Physics.

[110] Li Y, Hu J, Sari H, Xue S, Ma R, Kandarpa S, et al. A deep neural network for parametric image reconstruction on a large axial field-of-view PET. Eur J Nucl Med Mol Imaging. 2022. 10.1007/s00259-022-06003-4. 10.1007/s00259-022-06003-4 36326869

[111] Sari H, Eriksson L, Mingels C, Alberts I, Casey ME, Afshar-Oromieh A (2023). Feasibility of using abbreviated scan protocols with population-based input functions for accurate kinetic modeling of [(18)F]-FDG datasets from a long axial FOV PET scanner. Eur J Nucl Med Mol Imaging.

[112] Rowe SP, Pomper MG, Leal JP, Schneider R, Kruger S, Chu LC, et al. Photorealistic three-dimensional visualization of fusion datasets: cinematic rendering of PET/CT. Abdom Radiol (NY). 2022. 10.1007/s00261-022-03614-1. 10.1007/s00261-022-03614-1 35916942

[113] Chicklore S, Goh V, Siddique M, Roy A, Marsden PK, Cook GJ (2013). Quantifying tumour heterogeneity in 18F-FDG PET/CT imaging by texture analysis. Eur J Nucl Med Mol Imaging.

[114] Hatt M, Tixier F, Visvikis D, Cheze Le Rest C (2017). Radiomics in PET/CT: more than meets the eye?. J Nucl Med.

[115] Mayerhoefer ME, Materka A, Langs G, Haggstrom I, Szczypinski P, Gibbs P (2020). Introduction to radiomics. J Nucl Med.

[116] Cook GJR, Azad G, Owczarczyk K, Siddique M, Goh V (2018). Challenges and promises of PET radiomics. Int J Radiat Oncol Biol Phys.

[117] Zwanenburg A (2019). Radiomics in nuclear medicine: robustness, reproducibility, standardization, and how to avoid data analysis traps and replication crisis. Eur J Nucl Med Mol Imaging.

[118] Berenguer R, Pastor-Juan MR, Canales-Vázquez J, Castro-García M, Villas MV, Mansilla Legorburo F (2018). Radiomics of CT features may be nonreproducible and redundant: influence of CT acquisition parameters. Radiology.

[119] Sollini M, Cozzi L, Antunovic L, Chiti A, Kirienko M (2017). PET Radiomics in NSCLC: state of the art and a proposal for harmonization of methodology. Sci Rep.

[120] Desseroit MC, Tixier F, Weber WA, Siegel BA, Cheze Le Rest C, Visvikis D (2017). Reliability of PET/CT shape and heterogeneity features in functional and morphologic components of non-small cell lung cancer tumors: a repeatability analysis in a prospective multicenter cohort. J Nucl Med.

[121] Da-Ano R, Visvikis D, Hatt M (2020). Harmonization strategies for multicenter radiomics investigations. Phys Med Biol.

[122] Adachi T, Nagasawa R, Nakamura M, Kakino R, Mizowaki T (2022). Vulnerabilities of radiomic features to respiratory motion on four-dimensional computed tomography-based average intensity projection images: a phantom study. J Appl Clin Med Phys.

[123] Prenosil GA, Weitzel T, Furstner M, Hentschel M, Krause T, Cumming P (2020). Towards guidelines to harmonize textural features in PET: Haralick textural features vary with image noise, but exposure-invariant domains enable comparable PET radiomics. PLoS One.

[124] Zhang J, Knopp MI, Knopp MV (2019). Sparse detector configuration in SiPM digital photon counting PET: a feasibility study. Mol Imaging Biol.

[125] Gallach M, Mikhail Lette M, Abdel-Wahab M, Giammarile F, Pellet O, Paez D (2020). Addressing global inequities in positron emission tomography-computed tomography (PET-CT) for cancer management: a statistical model to guide strategic planning. Med Sci Monit.

[126] Surti S, Karp JS (2020). Update on latest advances in time-of-flight PET. Phys Med.

[127] Surti S, Karp JS (2021). Reconstruction-free positron emission imaging. Nat Photonics.

[128] Kuramoto M, Nakamori T, Kimura S, Gunji S, Takakura M, Kataoka J (2017). Development of TOF-PET using Compton scattering by plastic scintillators. Nucl Instrum Methods Phys Res A Accel Spectrom Detect Assoc Equip.

[129] Watts DP, Bordes J, Brown JR, Cherlin A, Newton R, Allison J (2021). Photon quantum entanglement in the MeV regime and its application in PET imaging. Nat Commun.

[130] Alkhorayef M, Sulieman A, Alsager OA, Alrumayan F, Alkhomashi N (2021). Investigation of using positronium and its annihilation for hypoxia PET imaging. Radiat Phys Chem.

[131] Shibuya K, Saito H, Nishikido F, Takahashi M, Yamaya T (2020). Oxygen sensing ability of positronium atom for tumor hypoxia imaging. Commun Phys.

[132] Moskal P, Dulski K, Chug N, Curceanu C, Czerwiński E, Dadgar M, et al. Positronium imaging with the novel multiphoton PET scanner. Sci Adv. 7:eabh4394. 10.1126/sciadv.abh4394. 10.1126/sciadv.abh4394 PMC11559468 34644101

[133] Moskal P, Kisielewska D, Curceanu C, Czerwiński E, Dulski K, Gajos A (2019). Feasibility study of the positronium imaging with the J-PET tomograph. Phys Med Biol.

[134] Shibuya K, Saito H, Tashima H, Yamaya T (2022). Using inverse Laplace transform in positronium lifetime imaging. Phys Med Biol.

[135] Schramm G. Reconstruction-free positron emission imaging: fact or fiction? Front Nucl Med. 2022;2:936091. 10.3389/fnume.2022.936091. 10.3389/fnume.2022.936091 PMC11440944 39354988

[136] Toussaint M, Lecomte R, Dussault JP (2020). Annihilation photon acolinearity with ultra-fast ToF-PET. 2020 IEEE Nuclear Science Symposium and Medical Imaging Conference (NSS/MIC).

